# Erythema Nodosum: A Consequence of Tuberculosis

**DOI:** 10.7759/cureus.4724

**Published:** 2019-05-23

**Authors:** Zain Rizvi, Tahir Iqbal, Aaesha Javed, Asjad Rizvi

**Affiliations:** 1 Internal Medicine, Shifa International Hospital, Islamabad, PAK; 2 Medicine, Islamabad Medical and Dental College, Islamabad, PAK

**Keywords:** tuberculosis, erythema nodosum, reactive arthritis, pulmonary, dermatology

## Abstract

Erythema nodosum is a form of panniculitis that presents as red lumps most commonly on the shins. It commonly presents due to tuberculosis, streptococcal infections, sarcoidosis, or can be drug related. This report describes the case of a young woman who presented with erythema nodosum and reactive arthritis; this was determined to be a sequelae of tuberculosis. Investigations were ordered as there was a high level of clinical suspicion for an underlying systemic cause for the presenting complaints. The systemic condition was determined to be tuberculosis due to the endemic environment and a positive tuberculin skin test.

## Introduction

Erythema nodosum (EN), a type of panniculitis, presents as red lumps most commonly on the shins and less commonly on the forearms and thighs. It is a well-known immunologic reaction to different stimuli such as drugs, infections, and malignant diseases. This painful condition of the subcutaneous fat typically presents with tender, erythematous, subcutaneous nodules of varying sizes.

The most common cause of EN worldwide is idiopathic (55%); frequently, particularly in children, it is associated with streptococcal infections [1]. Tuberculosis was historically an important cause of EN but the incidence has decreased dramatically at the present time; however, it must be excluded in developing countries [2, 3]. The incidence of EN presenting with tuberculosis is mostly considered to be rare as it is usually associated with other diseases [4, 5]. It should be noted that in countries where tuberculosis is an endemic disease, the prevalence of EN increases in direct correlation with the prevalence of tuberculosis [6, 7]. Apart from tuberculosis, other systemic causes like sarcoidosis and inflammatory bowel disease must be considered as they comprise 25% of all cases [8]. Furthermore, EN can be caused due to drug intake, especially sulphonamides and amoxicillin; this accounts for about 15% of presenting cases [8].

Erythema nodosum typically starts with a prodromal state, and this occurs one to three weeks before the onset of the painful, erythematous rash. This prodrome state can then lead to specific symptoms such as arthralgia with or without arthritis, weight loss, malaise, and fever. A patient presenting with arthritis-like symptoms and EN can be described as secondary due to tuberculosis; this is called Poncet’s disease, a rare presentation of tuberculosis [9]. Furthermore, the definitive diagnosis must rule out the mentioned causes according to their prevalence in the health care setting.

Hence, a patient with erythema nodosum presenting to primary care should have an extensive diagnostic evaluation to consider and rule out the numerous differential diagnoses, and especially in this case tuberculosis must be ruled out.

We describe a case of a young female who presented with erythema nodosum and bilateral ankle arthritis. Investigations showed a positive tuberculin skin test (TST) but no detectable tubercular infection upon further investigations. She was treated with anti-tuberculosis therapy with resolution of her symptoms.

## Case presentation

A 35-year-old Pakistani woman presented to our outpatient clinic with painful swollen ankle joints which had started three days ago. This was preceded by a painful, erythematous rash consisting of five nodules located on the anterior aspect of her right tibia, and four nodules on her left tibia, two months prior to the onset of ankle swelling. The patient did not report any other symptoms such as fever, fatigue, malaise, weight loss, dysuria or cough. There was no significant past medical history. She was prescribed oral prednisolone and topical Fucidin 2% ointment two months previously for her rash.

On physical examination, the respiratory and cardiovascular systems were normal. There was no apparent lymphadenopathy or hepatosplenomegaly. On inspection, both ankles were red, hot, and swollen, and limitation in movements was apparent. The nodules present on the tibia were poorly demarcated, 1-3 cm in diameter, and erythematous. On palpation, the nodules and area surrounding them were tender.

Laboratory tests revealed a normal blood count, with the exception of a raised erythrocyte sedimentation rate (ESR) which was 29 mm in the first hour. Furthermore, liver function tests (alanine transaminase (ALT), aspartate aminotransferase (AST), alkaline phosphatase (ALP), glucose tolerance test (GTT), bilirubin), antistreptolysin O (ASO) titers, and renal function (creatinine, urea) were not deranged. Thyroid function tests (free T3, free T4 and thyroid-stimulating hormone (TSH)) were normal. Chest radiography was performed and was a normal study. A two-step tuberculosis skin test (TST, Mantoux) was performed on the patient’s left arm and patient was initially prescribed analgesics for her ankle pain. The patient returned after 72 hours for re-evaluation and she presented with a positive TST with an induration of 16 mm (positive >10 mm in endemic regions), alongside reduction in pain but with persistence of the rash.

In clinical context and due to the geographical prevalence of the disease, reactive arthritis and erythema nodosum secondary to tuberculosis was diagnosed and patient followed up with a tuberculosis specialist for further evaluation and management. Family members of the patient were screened for exposure to tuberculosis; there were no positive results on TST.

**Figure 1:**
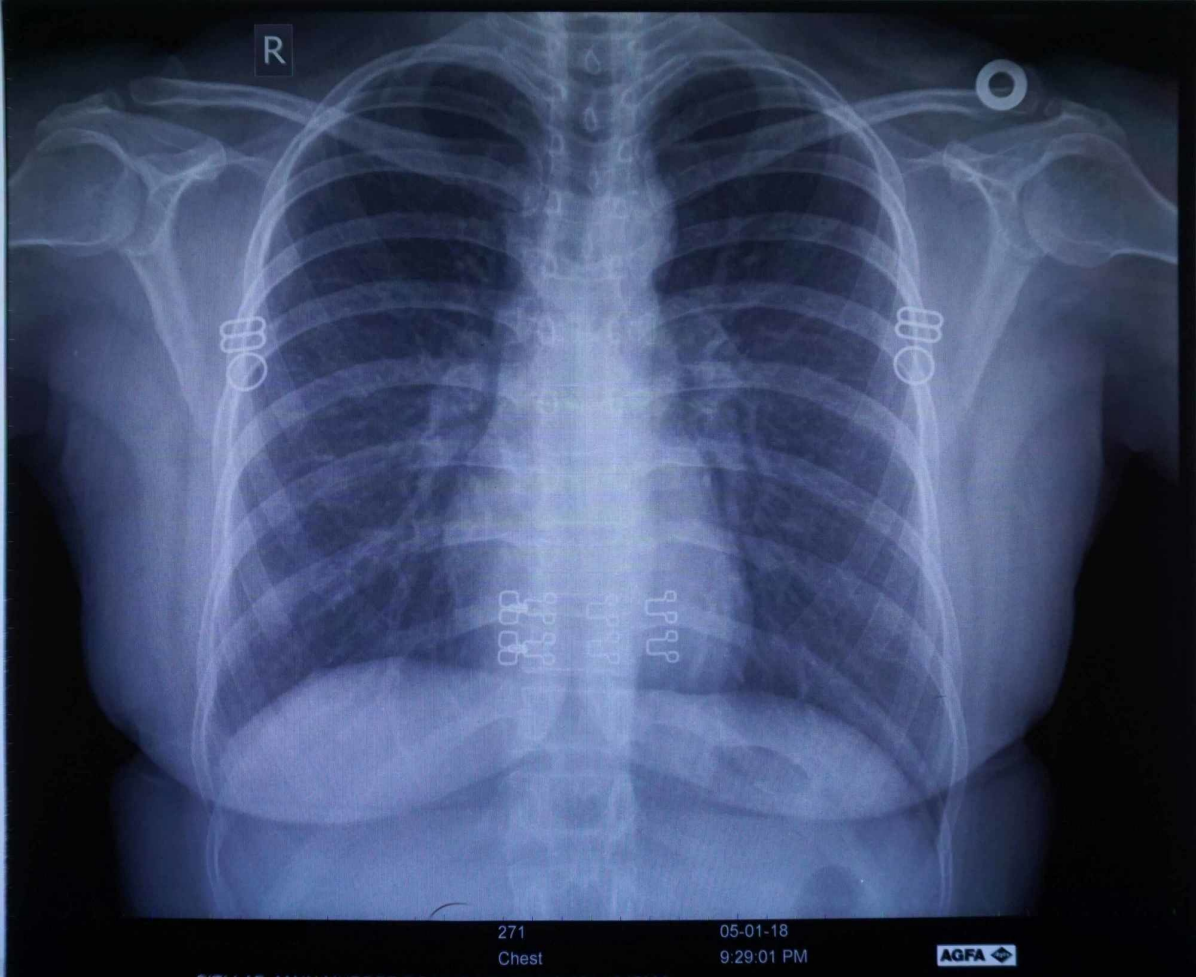
Chest Radiography of the Patient

Imaging studies did not show any positive signs of tuberculosis (Figure 1) and due to no presenting respiratory complaints, no cultures were sent for diagnosis of tuberculosis. The diagnosis was considered despite lack of any detection on imaging or confirmation by culture as the TST tested positive twice by an increasing induration and due to the fact that Pakistan is a tuberculosis endemic country. The patient was advised to commence anti-tuberculosis therapy in the following manner: six months of isoniazid/rifampicin/ethambutol/pyrazinamide combined therapy, followed by three months of isoniazid/rifampicin therapy. Upon subsequent visits the patient’s rash had disappeared but her TST was positive consistently.

## Discussion

Erythema nodosum (EN) is a delayed type IV hypersensitivity response to different stimuli such as infections, drugs, autoimmune diseases, or it can be idiopathic. The patient’s country of origin and residence should be considered as it is linked to certain diseases that are endemic in countries [8]. The incidence of EN depends on the country due to its nature of being associated with certain diseases like tuberculosis, but worldwide it has an incidence of one to five per 100,000 persons, with an increased predisposition to females aged between 20-40 years [2, 3].

The majority of recent studies provide evidence of the association between erythema nodosum and tuberculosis [3, 7, 8]. Infrequently it has been suggested in studies that erythema nodosum is not associated with tuberculosis [4, 5], but due to new mounting evidence, there is a strong inclination towards the association. This association is most apparent in regions where tuberculosis is endemic; two Asian studies showed that there is a positive correlation of tuberculosis patients having erythema nodosum and vice versa. These studies support the association of tuberculosis leading to erythema nodosum in endemic areas [10, 11]. Furthermore, the study by Chen et al. suggests that tuberculosis contributes to the pathogenesis of erythema nodosum and may be used as a predictor for primary extra-pulmonary tuberculosis. Other studies suggest primary tuberculosis is the only form that leads to erythema nodosum [6].

It should be noted that a detectable active infection of tuberculosis is not always present; research justifies the approach to investigate patients for tuberculosis when presenting with erythema nodosum, especially in endemic regions or due to their country of origin. The investigations performed should include a two-step tuberculosis skin test (TST), chest radiography, and, if possible, acid-fast bacilli sputum culture [6].

Erythema nodosum presenting with reactive arthritis and a positive TST is a rare association, and this is sometimes called Poncet’s disease [9]. The arthritic joints should ideally be diagnosed based on joint aspiration and analysis to confirm there is no septic nature to the pain. Reactive arthritis due to tuberculosis is only verified if the analysis demonstrates aseptic fluid. However, in this case the arthritis was diagnosed clinically as the joints were painful, warm and resulted in restriction of movements.

According to WHO guidelines regarding tuberculosis in an endemic country, the recommendation is to treat using the regimen for 9-12 months. For six months all four drugs are given and for the remaining three to six months, only two drugs are given (isoniazid, rifampicin). For a patient presenting with a positive TST and erythema nodosum, even with no positive focus of tuberculosis infection, it is advised to provide proper anti-tuberculosis therapy as the disease may be too minuscule to be detected by conventional methods [12]. Our patient from Pakistan, a tuberculosis endemic country, presented with erythema nodosum, reactive arthritis, and a positive TST, and this allowed enough evidence to prescribe anti-tuberculosis therapy. The patient’s symptoms improved drastically and there was complete resolution within the first month of the nine month regimen.

## Conclusions

The diagnosis of the primary etiology of erythema nodosum is of paramount importance as there are therapeutic implications attached to the numerous causes for the patient and for close contacts. Our patient was found to have an underlying tuberculosis infection that when treated adequately caused the patient's presenting complaints to subside. Due to migration from endemic areas, increased incidence of tuberculosis, HIV, and increased drug usage have emerged as important causes of erythema nodosum in non-endemic areas. These factors should be considered by clinicians and should be ruled out as underlying causes in patients who present with erythema nodosum, especially if they have any risk factors for it.
